# Ruxolitinib in conjunction with the HLH-94 protocol for Epstein-Barr virus-related hemophagocytic lymphohistiocytosis in the intensive care unit: A case report

**DOI:** 10.1097/MD.0000000000025188

**Published:** 2021-03-19

**Authors:** Zoufang Huang, Jiangbo Xie

**Affiliations:** a Department of Hematology; b Department of Intensive Care Unit, the First Affiliated Hospital of Gannan Medical University, Jiangxi, China.

**Keywords:** Epstein-Barr virus, hemophagocytic lymphohistiocytosis, ruxolitinib

## Abstract

**Rationale:**

The HLH-94 protocol is a standard induction treatment for hemophagocytic lymphohistiocytosis. However, about 30% of patients may not respond. Ruxolitinib has been clinically proven to be an effective treatment for hemophagocytic lymphohistiocytosis (HLH).

**Patient concerns:**

A previously healthy 14-year-old girl presented to the local hospital with a 4-day history of persistent fever and sore throat.

**Diagnosis:**

Clinical and laboratory tests revealed fever >38.5°C, hepatosplenomegaly, pancytopenia, hypertriglyceridemia, hypofibrinogenemia, hyperferritinemia, and an elevated interleukin-2 receptor level.

**Intervention:**

This patient was treated with ruxolitinib and the HLH-94 protocol.

**Outcomes:**

The patient's clinical and some laboratory indices improved. Unfortunately, vital signs such as respiratory function and consciousness did not improve.

**Lessons:**

This case report highlights the effect of using ruxolitinib in conjunction with the HLH-94 protocol. However, safety evaluation of this regimen was not performed because critically ill patient died too fast.

## 1 Introduction

Hemophagocytic lymphohistiocytosis (HLH) is a life-threatening disorder characterized by an excessive inflammatory response mediated by hyperactivation of T cells and antigen-presenting cells. Epstein-Barr virus (EBV) is a common triggering factor for HLH. EBV-associated HLH (EBV-HLH) can progress rapidly to multiorgan dysfunction. Symptoms of HLH include persistent pyrexia, pancytopenia, hepatosplenomegaly, elevated lactate dehydrogenase, serum ferritin, and triglyceride levels, and decreased fibrinogen.^[1,2]^ The HLH-94 protocol is a standard induction treatment for HLH, consisting of dexamethasone and etoposide.^[3]^ However, about 30% of patients may not respond.^[4]^ Ruxolitinib has been clinically proven to be effective to treat HLH.^[5–7]^ Thus, we use ruxolitinib in conjunction with the HLH-94 protocol as the first-line treatment.

Here we report a case of a patient with newly diagnosis EBV-triggered HLH who was critically ill and experienced improvement after ruxolitinib in conjunction with the HLH-94 protocol.

### 1.1 Consent statement

The patient's family had provided informed consent for the publication of this case report.

## 2 Case report

A previously healthy 14-year-old girl presented to the local hospital with a 4-day history of persistent fever and sore throat. After taking acetaminophen for 2 days, she felt worse and complained of extremity myalgia. Laboratory tests revealed pancytopenia (white blood cell [WBC] count, 2.69 × 10^9^/L; neutrophil count, 1.96 × 10^9^/L; hemoglobin, 93 g/L; platelet count, 34 × 10^9^/L), abnormal liver function (aspartate aminotransferase, 300 units/L [normal: <40 units/L]; alanine aminotransferase, 111 units/L [normal: <35 units/L]; total bilirubin, 102 μmol/L [normal: <21 μmol/L]; albumin, 27.6 g/L [normal: 40–55 g/L], and creatinine, 247 μmol/L [normal: 41–73 μmol/L]). The patient was diagnosed with multiorgan failure and admitted to our hospital.

Meropenem and vancomycin treatment was initiated within 3 days of admission, and a chest computed tomography (CT) scan revealed pneumonia. On hospital day 2, thrombotic thrombocytopenic purpura was suspected. Dexamethasone 10 mg/day, intravenous immunoglobulin 0.4 g/kg/day, and plasmapheresis were administered. Her condition worsened, with a persistent fever of 38.5 to 39.8°C and rapid heart rate of >140 bpm. Her blood pressure was about 90/60 mm Hg, and she was supported with 1.6 mg/hour norepinephrine. She received mechanical ventilation because of respiratory failure. Platelets and fibrinogen were not elevated after an infusion. She had persistent epistaxis and coma on hospital day 3. Rheumatological, autoimmune, and oncological workups revealed no positive results (Table 1). EBV polymerase chain reaction revealed 1.03 × 10^6^ copies/ml. Metagenomic next-generation sequencing (NGS) was positive for EBV but negative for bacteria, fungus, parasites, and *Mycobacterium*. Lactate dehydrogenase (LDH) was 3560 U/L. The patient met all 8 criteria for HLH, that is, fever >38.5°C, hepatosplenomegaly (based on Doppler ultrasound and abdominal CT), pancytopenia (WBC count, 0.67 × 10^9^ /L, hemoglobin, 67 g/L; platelet count, 2 × 10^9^ /L), hypertriglyceridemia (5.89 g/L), hypofibrinogenemia (0.72 g/L), hyperferritinemia (100,096 ng/ml), elevated interleukin-2 receptor level (40,740 U/ml), and hemophagocytosis observed on a bone marrow biopsy specimen. Genetic testing was performed and was negative for any known mutations causing HLH.

**Table 1:** The patient's laboratory results.

Infectious
Blood cultures: negative
COVID-19 PCR: negative
EBV PCR: 1.5 × 10^6^ copies/ml
EBV NGS: positive
HIV antibody: negative
Hepatitis B surface antigen: negative
Hepatitis B surface IgG: positive
Hepatitis B core IgG: negative
Hepatitis C antibody: negative
Widal reaction: negative
Weil-Felix assay: negative
Immunologic
CD3+: 640/μl (normal: 770–2,860/μl)
CD4+: 308/μl (normal: 414–1,440/μl)
CD8+: 328/μl (normal: 238–1,250/μl)
CD4/CD8: 0.94 (normal: 0.7–2.87)
Rheumatologic
C3: 53.7 mg/dl (normal: 70–140 mg/dl)
C4: 38.2 mg/dl (normal: 10–40 mg/dl)
Rheumatoid factor:18.1 IU/ml (normal,: <25 IU/ml)
Antibody spectrum of anti-ENA peptide: negative
Antinuclear antibody: negative
ADAMTS13 activity: normal
Oncologic
CT chest, abdomen biopsy negative for malignancy
Bone marrow biopsy negative for leukemia

EBV = Epstein-Barr virus, NGS = next-generation sequencing, PCR = polymerase chain reaction.

**Figure 1:**
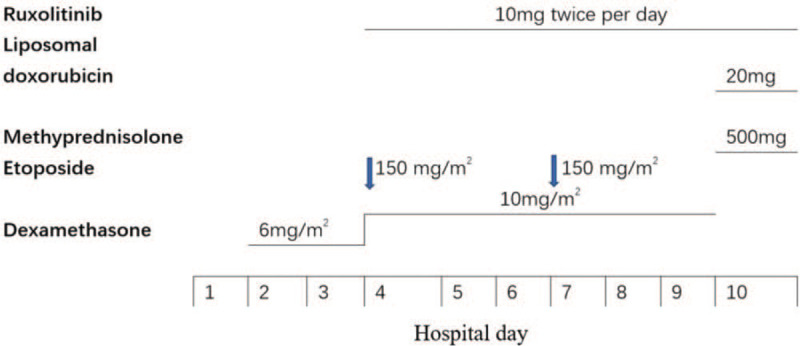
The treatment processes.

**Figure 2:**
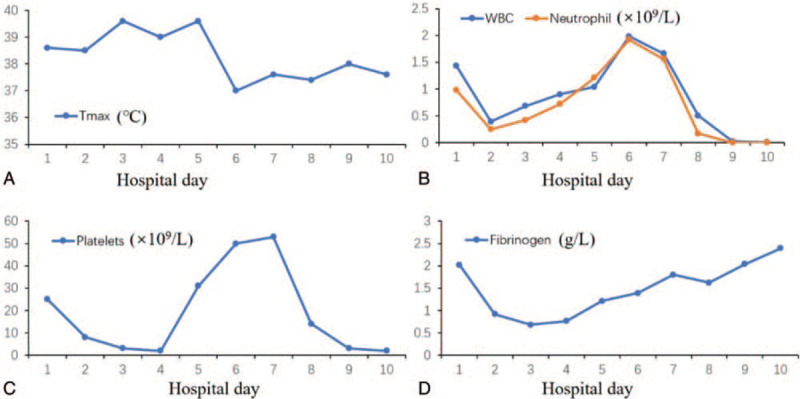
The patient's treatment response. The temperature returned to normal and the heart rate dropped to about 105 bpm after 20 hours (A). Improvements were seen in several indices, including the white blood cell count (B) platelet count (C), and fibrinogen level (D).

After confirming the diagnosis on hospital day 4, ruxolitinib (10 mg twice per day) was administered in conjunction with the HLH-94 protocol (Fig. 1). Her temperature returned to normal and the heart rate dropped to about 105 bpm after 20 hours (Fig. 2A). Her blood pressure returned to normal after administering 0.2 mg/hour norepinephrine. Improvements were seen in several indices, including the WBC count (Fig. 2B), platelet count (Fig. 2C), and fibrinogen level (Fig. 2D). Her platelet count increased to 53 × 10^9^ /L after infusion. Her fibrinogen level returned to normal, and there was a normal prothrombin time and activated partial thromboplastin. D-dimer decreased from 125 mg/L to 2.5 mg/L. EBV dropped to 2.35 × 10^4^ copies/ml on hospital day 6. However, LDH and serum ferritin levels increased to 6,442 U/L and 42,897 ng/ml on hospital day 7, respectively. She remained in a coma on mechanical ventilation with high flow oxygen. On hospital day 8, her blood cell count decreased again (WBC count, 0.02 × 10^9^ /L; hemoglobin, 80 g/L; platelet count, 2 × 10^9^/L). On hospital day 10, we utilized the DEP protocol (20 mg liposomal doxorubicin, 100 mg etoposide, and 500 mg methylprednisone) rather than the HLH94 protocol. She died of cardiac arrest at 11 pm that day.

## 3 Discussion

HLH is a fatal clinical syndrome and no optimal treatment is available for HLH, the mortality rate for which exceeds 40% in children and adults even when following the standard HLH-94 protocol.^[2,8]^ Clinicians have tried other combinations of agents. Ruxolitinib is a promising agent due to its powerful calming effect on the cytokine storm.^[9]^ Ruxolitinib is a Janus kinase 1/2 inhibitor that suppresses the transmission of cytokine-induced signals. It suppresses cytokine signaling pathways, such as those of interferon (IFN)-γ and interleukin-2. In murine models of HLH, mice who received ruxolitinib had improved survival, due to reversal of pancytopenia and splenomegaly, and reductions in proinflammatory cytokines and the number of CD8+ T cells.^[10,11]^ Ruxolitinib also reduces the severity of inflammation-associated anemia, and the number and activation status of T cells and neutrophils in primary and secondary HLH murine models, but only the IFN-γ-neutralizing antibody reduces anemia severity.^[12]^ Ruxolitinib increased the apoptotic potential of CD8+ T cells in a murine model and primary patient samples, which were resistant to dexamethasone.^[13]^

In the human body, ruxolitinib has been demonstrated to exert a dramatic effect in some acute inflammatory syndromes, including acute graft-vs-host disease,^[14]^ steroid-refractory cytokine release syndrome,^[15]^ and COVID-19 with severe systemic hyperinflammation.^[16]^ As a single drug, ruxolitinib has been used in salvage therapy for HLH, and exerted a powerful effect in several case reports.^[17–21]^ Similar to what we observed in our case, the first reported case was a 38-year-old woman diagnosed as secondary HLH caused by an EBV infection. This patient exhibited improving disease markers but died eventually.^[17]^ The improving markers included ferritin, fibrinogen, and LDH concentrations, but not pancytopenia. However, a 26-year-old woman diagnosed with treatment-refractory HLH caused by EBV and acute hepatitis C virus infection achieved completely recovery after the use of ruxolitinib.^[19]^ In a series of children cases reported in China, the authors reported poor response to ruxolitinib alone in EBV-HLH when compared with other causes of HLH.^[20]^ The authors suggested that ruxolitinib might not be able to eradicate EBV because EBV does not solely rely on JAK-STAT pathway to cause HLH. Therefore, combination with other drugs is needed.

Small series have reported improved clinical outcomes using ruxolitinib in conjunction with the HLH-94 protocol or chemotherapy.^[6,22,23]^ In a multicenter, nonrandomized, phase II trial for refractory/relapsed HLH (NCT03533790), 54 patients received ruxolitinib combined with the doxorubicin-etoposide-methylprednisolone regimen for 2 weeks. Excitingly, in the patients who had previously received the DEP regimen but showed no improvement, 7 of 12 (58·3%) exhibited a partial response.^[23]^ A pilot trial with first-line ruxolitinib as a single agent reported responses in 5 patients (NCT02400463), and the 2-month overall survival rate was 100%.^[7]^ However, EBV-associated HLH was excluded from that trial. The most exciting outcome of ruxolitinib as a single agent for EBV-associated HLH was a 100% response rate (complete response, 75%, partial response, 25%) in a Chinese trial (ChiCTR2000029977).^[5]^ Three cases treated with first-line ruxolitinib plus HLH-94 protocol showed rapid response and no obvious adverse effects.^[6]^ For our patient who was treated with ruxolitinib plus HLH-94 protocol for the first line, clinical and some laboratory indices improved. Unfortunately, the vital signs, such as respiratory function and consciousness, did not improve.

## Author contributions

**Resources:** Jiangbo Xie.

**Writing – original draft:** Zoufang Huang.

**Writing – review & editing:** Jiangbo Xie.
